# Effects of the COVID-19 pandemic on the outcomes of HIV-exposed neonates: a Zimbabwean tertiary hospital experience

**DOI:** 10.1186/s12887-023-04473-5

**Published:** 2024-01-05

**Authors:** Hannah Gannon, Elizabeth Chappell, Deborah Ford, Diana M Gibb, Anesu Chimwaza, Ngoni Manika, Catherine J Wedderburn, Zivai Mupambireyi Nenguke, Frances M Cowan, Tom Gibb, Andrew Phillips, Angela Mushavi, Felicity Fitzgerald, Michelle Heys, Simbarashe Chimhuya, Mutsa Bwakura-Dangarembizi

**Affiliations:** 1 UCL Great Ormond Street Institute of Child Health, University College London, London, UK; 2 MRC Clinical Trials Unit at UCL, London, UK; 3 Ministry of Health and Child Care, Harare, Zimbabwe; 4 Department of Paediatrics and Child Health, University of Cape Town, Cape Town, South Africa; 5 Centre for Sexual Health and HIV/AIDS Research (CeSHHAR), Harare, Zimbabwe; 6 Liverpool School of Tropical Medicine, Liverpool, UK; 7 Picturing Health, London, UK; 8 Institute for Global Health, UCL, London, UK; 9 Department of Infectious Disease, Imperial College London, London, UK; 10 Child and Adolescent Health Unit, Faculty of Medicine and Health Sciences, University of Zimbabwe, Harare, Zimbabwe

**Keywords:** COVID-19, HIV, Neonates, Maternal health, Vertical transmission

## Abstract

**Introduction:**

The COVID-19 pandemic has globally impacted health service access, delivery and resources. There are limited data regarding the impact on the prevention of mother to child transmission (PMTCT) service delivery in low-resource settings. Neotree (www.neotree.org) combines data collection, clinical decision support and education to improve care for neonates. Here we evaluate impacts of COVID-19 on care for HIV-exposed neonates.

**Methods:**

Data on HIV-exposed neonates admitted to the neonatal unit (NNU) at Sally Mugabe Central Hospital, Zimbabwe, between 01/06/2019 and 31/12/2021 were analysed, with pandemic start defined as 21/03/2020 and periods of industrial action (doctors (September 2019-January 2020) and nurses (June 2020-September 2020)) included, resulting in modelling during six time periods: pre-doctors’ strike (baseline); doctors’ strike; post-doctors’ strike and pre-COVID; COVID and pre-nurses’ strike; nurses’ strike; post nurses’ strike. Interrupted time series models were used to explore changes in indicators over time.

**Results:**

Of 8,333 neonates admitted to the NNU, 904 (11%) were HIV-exposed. Mothers of 706/765 (92%) HIV-exposed neonates reported receipt of antiretroviral therapy (ART) during pregnancy. Compared to the baseline period when average admissions were 78 per week (95% confidence interval (CI) 70–87), significantly fewer neonates were admitted during all subsequent periods until after the nurses’ strike, with the lowest average number during the nurses’ strike (28, 95% CI 23–34, p < 0.001). Across all time periods excluding the nurses strike, average mortality was 20% (95% CI 18–21), but rose to 34% (95% CI 25, 46) during the nurses’ strike. There was no evidence for heterogeneity (p > 0.22) in numbers of admissions or mortality by HIV exposure status. Fewer HIV-exposed neonates received a PCR test during the pandemic (23%) compared to the pre-pandemic periods (40%) (RR 0.59, 95% CI 0.41–0.84, p < 0.001). The proportion of HIV-exposed neonates who received antiretroviral prophylaxis during admission was high throughout, averaging between 84% and 95% in each time-period.

**Conclusion:**

While antiretroviral prophylaxis for HIV-exposed neonates remained high throughout, concerning data on low admissions and increased mortality, similar in HIV-exposed and unexposed neonates, and reduced HIV testing, suggest some aspects of care may have been compromised due to indirect effects of the pandemic.

**Supplementary Information:**

The online version contains supplementary material available at 10.1186/s12887-023-04473-5.

## Introduction

The COVID-19 pandemic has disrupted health systems globally, with an impact on health service access, delivery and resources [1–3]. Mitigation strategies, including nationwide lockdowns, to limit the spread of the virus have also, in turn, had a significant negative impact with disruption of supply chains and life-saving health services, including antenatal care and HIV programmes [1, 2]. The importance of antenatal care and screening in the prevention of mother to child transmission (PMTCT) of HIV has been well documented and encompass key prevention strategies within global HIV programmes [4]. Although there have been gains in neonatal survival globally, eastern and southern Africa remain the regions with the highest burden of both neonatal mortality and HIV [4, 5]. In Zimbabwe in 2019 91% of pregnant women living with HIV had received antiretroviral therapy (ART), an increase from 30% in 2010, and early infant diagnosis (EID) had risen to 55.7% from 9.2% in 2010 [6]. However, external crises, such as the COVID-19 pandemic, threaten these gains, with risks of significant increases in maternal and neonatal deaths [7]. Zimbabwe is no exception and the pandemic has further increased the burden on Zimbabwe’s under-resourced and over-crowded health system, a health system which has already suffered from humanitarian and political crises [8].

There are limited data regarding the impact of the COVID-19 pandemic on PMTCT delivery in low resource settings (LRS) and health outcomes for these mothers and neonates. At the beginning of the pandemic modelling had suggested that a disruption of six months of ART could result in a 1.6 times increase in babies born with HIV in sub-Saharan Africa and that within Zimbabwe a three month disruption in PMTCT services could result in a relative increase of new HIV child infections by 1.53 [9]. The Global Fund reported widespread disruption of HIV, TB and Malaria service delivery impacting up to 75% of programmes globally [1]. The first case of COVID-19 in Zimbabwe was reported on the 21st March 2020 and a national level lockdown commenced on the 30th March 2020 with all but essential workers able to travel, all informal markets and schools closed, public transport suspended and shops only open for four hours a day. This initial national level lockdown was lifted in September 2020 but further lockdowns followed each of the subsequent waves of COVID-19.

Neotree is a Wellcome Trust funded (www.neotree.org) digital health platform aiming at improving newborn survival in low-resource settings. It offers immediate data capture, clinical decision support and data-driven quality improvement. It has been instituted at Sally Mugabe Central Hospital’s neonatal unit (NNU) since November 2018 as the admission and discharge documentation for the NNU [10]. Sally Mugabe Central hospital’s NNU, Harare, Zimbabwe, is the largest of five tertiary centres in Zimbabwe. An estimated 12,000 neonates are delivered annually and the NNU often functions at 140% of its 100 cot-bed capacity. The results of the development and pilot implementation of Neotree are reported elsewhere [10–12]. Previous analysis of Neotree data (from 1st June 2019 to 25th September 2020) showed admissions to the NNU at Sally Mugabe Central Hospital were not directly impacted during the initial months of the COVID-19 pandemic, but dropped substantially during a nurses’ strike in response to challenges exacerbated by the pandemic [13]. This study extends this timeline and aims to specifically examine the impact of COVID-19 on the outcomes of admitted HIV-exposed neonates, in a tertiary neonatal unit in Zimbabwe.

This analysis was part of a wider mixed methods study evaluating the impact of COVID-19 on PMTCT services in Zimbabwe including analysis of aggregate population and programme level health data [14], qualitative interviews and the development of educational materials for healthcare professionals, pregnant women and the wider community. Here we present the granular data from Sally Mugabe Central Hospital, the largest neonatal unit in Zimbabwe.

## Methods

### Study setting

The Neotree database captures individual level data on all neonates admitted to Sally Mugabe Central Hospital’s NNU since implementation (November 2018); this includes clinical observations, HIV testing, maternal demographics and outcomes. Data are captured on admission and then again at discharge and these two separate datasets are matched using a unique identifier: the Neotree ID.

### Current diagnosis and management of HIV-exposed neonates in Sally Mugabe Central Hospital

At Sally Mugabe Central Hospital the NNU follows the national guidance on the testing and management of HIV-exposed neonates. This guidance was introduced in 2016, to improve the identification of infants at highest risk for early disease progression, and recommends testing for early infant diagnosis (EID) by nucleic acid testing within the first two days of life [15]. If positive the neonate should be commenced on appropriate ART without delay and a second specimen taken to confirm the initial positive virological test. If negative, they should be re-tested at 4–6 weeks of age. HIV-exposed neonates should be commenced on antiretroviral prophylaxis, if assessed as low-risk they should receive Nevirapine (NVP) alone, HIV-exposed neonates assessed as high-risk should receive both NVP and Zidovudine (AZT).

A high-risk neonate is defined as follows:

- A neonate whose mother has a high viral load > 1000 copies/ml during the last four weeks before delivery.
- A neonate born to a woman living with HIV who has received less than 4 weeks of ART at the time of delivery.
- A neonate born to a woman newly diagnosed with HIV during labour, delivery or postpartum.

This guidance is followed within Sally Mugabe Central Hospital’s NNU when resource supplies for testing and management are available.

We considered 4 indicators:

1. The number of neonates admitted by HIV status.
2. The proportion of admitted neonates who died prior to discharge by HIV status.
3. The proportion of HIV-exposed neonates tested for HIV during admission.
4. The proportion of HIV-exposed neonates receiving antiretroviral prophylaxis at/by discharge.

Indicators 1 and 2 were summarised overall and by HIV exposure status (HIV-exposed neonates vs. not known to be HIV-exposed). Indicators 2–4 were restricted to neonates for whom matched outcome data were available.

### Definition of HIV exposure

Neonates were defined as HIV-exposed if they met one or more of the following criteria:

- Maternal ART use documented during pregnancy.
- Maternal HIV PCR test positive.
- Admission or discharge diagnosis as HIV-exposed.
- Cause of death as HIV-exposed.
- Neonatal PCR test during admission.
- NVP or AZT at admission or discharge.

### Statistical analysis

Data collected by Neotree between the 1st June 2019 and 31st December 2021 were analysed, with data preceding the COVID-19 pandemic used as a comparison. Two healthcare provider (HCP) strikes took place in Zimbabwe during the time-period of interest. Firstly, a doctors’ strike between 3rd September 2019 to 22nd January 2020, and secondly a nurses’ strike from 17th June 2020 to 9th September 2020. Based on the two periods of healthcare provider industrial action and the start of the COVID-19 pandemic (defined as the 21st March, the date of the first reported COVID-19 case in Zimbabwe), six distinct time periods were considered.

1. Before doctors’ strike (prior to COVID pandemic).
2. During the doctors’ strike (prior to COVID pandemic).
3. Doctor’s strike to start of COVID pandemic (prior to COVID pandemic).
4. Start of COVID-pandemic to nurses’ strike (during the COVID pandemic).
5. Nurses’ strike (during the COVID pandemic).
6. After the nurses’ strike (during the COVID pandemic).

Interrupted time series models with weekly (indicators one and two) or fortnightly (indicators three and four, due to the smaller number) data windows were used to explore changes over time. The mean number/proportion in each time period was estimated and compared to the period prior to the doctor’s strike. Quasi-poisson regression models accounting for overdispersion were then used, with an offset of the logarithm of total number of admissions per week for indicator two and of the number of HIV-exposed neonates admitted for indicators three and four to further explore trends over time [16]. Models were parameterised to allow a change in level and different slope in each time period; estimates were reported as the change over time in each time period and the relative change at the start of each time period compared to the end of the previous one. Differences by HIV exposure status (indicators 1 and 2 only) were explored using interaction terms with each of the level change and slope parameters. Autocorrelation was assessed by examining plots of residuals and the autocorrelation function, with no evidence found for any of the models. The analysis had approximately 90% power to detect each of a 15% reduction in the number of admissions and a 20% increase in mortality during the COVID pandemic. Statistical analyses were conducted using Stata version 17.0 (StataCorp, College Station, TX, USA).

### Missing values

Admission and discharge forms were matched using unique Neotree identity numbers. The matching algorithm uses a combination of this unique ID and the date of birth to accurately match. Neonates who did not have a complete set of matched admission and discharge forms were included in the analysis of the number of admissions, but excluded from analyses of the other outcomes.

### Research ethics

This study formed part of a wider mixed methods ViiV funded project; *“Evaluation of the Impact of the COVID-19 Pandemic on Provision and Uptake of Services for the Prevention of Mother-to-child Transmission of HIV and Syphilis in Zimbabwe”* and received ethics approval from the Medical Research Council of Zimbabwe MRCZ/A/2682. The Neotree pilot study received approval from Sally Mugabe Central Hospital Research Ethics Committee (Reference number HCHEC070618/58), University College London Ethics Committee (5019/004), Biomedical Research and Training Institute (AP148/18), the Medical Research Council of Zimbabwe MRCZ/A/2570 and the Electronic Health Records Department of the Zimbabwe Ministry of Health and Child Care. Neotree follows international and local precedent for collection of pseudonymised data for the purposes of epidemiological surveillance and service evaluation such as the neonatal UK/Australia/New Zealand Badgernet system, or the WHO-led District Health Information Software (DHIS). The need to obtain informed consent was waived by each of the ethical boards; the Medical Research Council of Zimbabwe, University College London Ethics Committee, Biomedical Research and Training Institute and Sally Mugabe Central Hospital’s Ethics committee, as we collected only pseudonymised data routinely documented for clinical care.

## Results

Across the whole study period, 8,333 babies were admitted to the neonatal unit; 6,005 (72%) were born within the hospital. In total, 904 (11%) were classified as HIV-exposed, of whom 706/765 (92%), with available data, were born to mothers reported to have received ART during pregnancy; 381/655 (58%), where timing was known, initiated ART prior to or during the first trimester. The majority, 7,208 (87%), of neonates were < 24 h of age at admission. Among 7,123 (85%) neonates with matched outcome data, the median duration of admission was four days (interquartile range (IQR) 2, 7). Table 1 shows the baseline demographics of the admitted neonates.

**Table 1:** Admission demographics and outcome

	N = 8,333 n(%) or median (IQR)
Place of birth (n = 7,976/8,333; 95%)	
Within Sally Mugabe Central Hospital	6,005 (75%)
Not in Sally Mugabe Central Hospital	1,971 (25%)
**HIV-exposed**	904 (11%)
**Maternal ART use in pregnancy (n = 765/904; 87%)**	706 (92%)
**Known timing of ART initiation (n = 706/765; 92%)**	564 (80%)
Started first trimester or earlier	381 (67%)
Started second trimester	62 (11%)
Started third trimester, up to 1 month before delivery	66 (12%)
Started less than 1 month before delivery	55 (10%)
**Birth weight (grams) (n = 7,783/8,333; 93%)**	2660 (1800,3200)
**Age at admission (n = 8,282/8,333; 99%)**	
<24 h	7,208 (87%)
24–48 h	568 (7%)
48–72 h	244 (3%)
72 h – 7 days	244 (3%)
>7 days	18 (< 0.5%)
**Duration of admissions, days**	4 (2, 7)
**Outcome among matched records (n = 7,123/8,333; 85%)**	
Discharged	5,596 (79%)
Died	1,509 (21%)
Transferred to another hospital	6 (< 0.5%)
Transferred to another ward	6 (< 0.5%)
Absconded	6 (< 0.5%)

*“n =* *n/n (%)” for each variable represents the number with complete data; data are complete where the number and proportion missing is not stated*

### Indicator 1: admissions

**Fig. 1:**
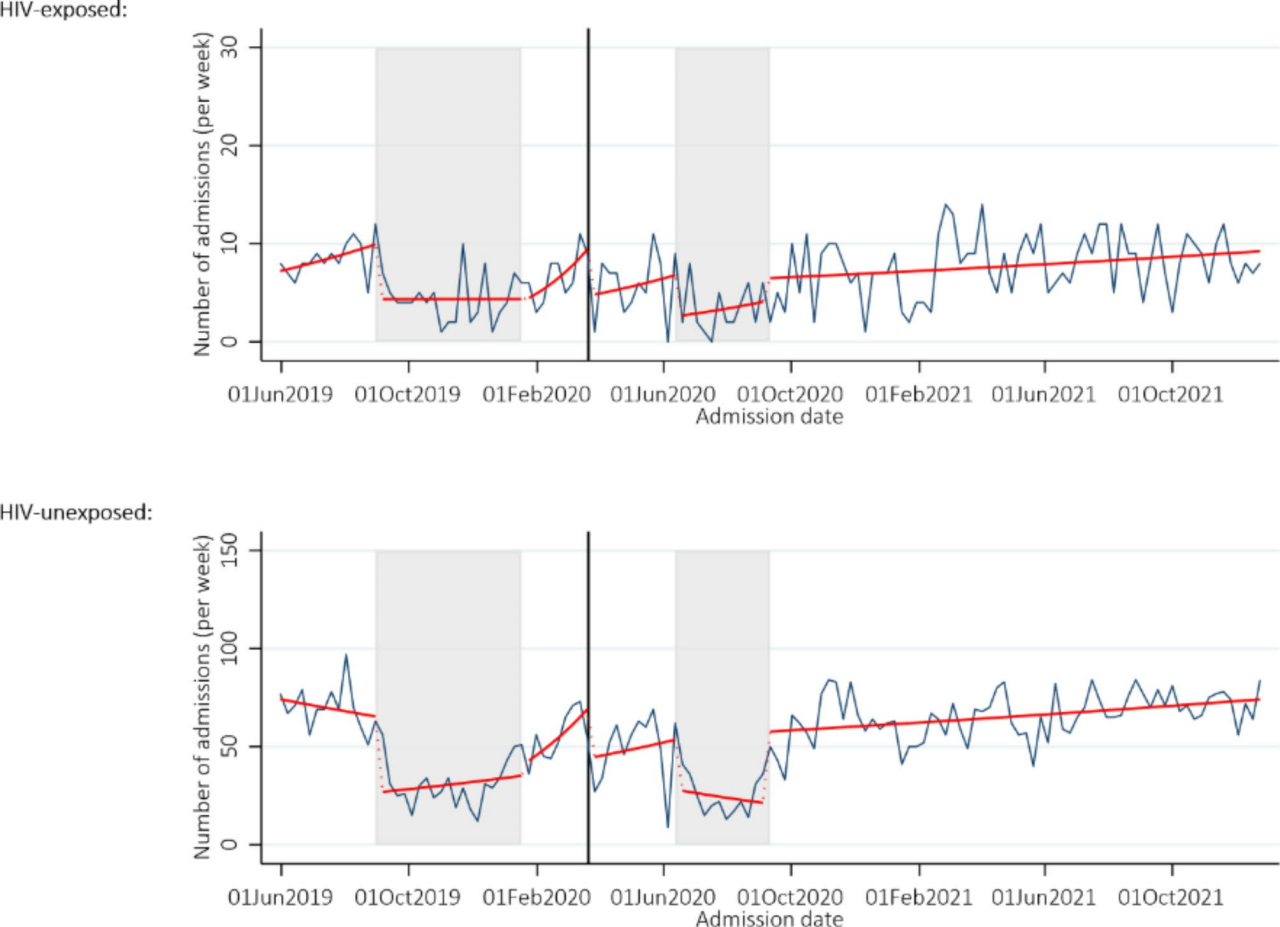
Admissions by HIV exposure. *Note:* The grey shaded areas represent the doctors and nurses strike, and the solid black vertical line represents the start of the COVID-19 pandemic in Zimbabwe. The blue line shows the number of admissions per week, and the red line the number from the fitted model allowing a change in slope and level in each time period

In the period prior to the doctors’ strike, the mean number of admissions per week was 78 (95% confidence interval (CI) 70–87) which fell over time (rate ratio (RR) 0.99 per week, 95% CI 0.97–1.02, *p* = 0.65). There was a small but not statistically significant drop in the number of admissions at the start of the COVID pandemic (RR 0.72, 95% CI 0.49–1.07, *p* = 0.10). Compared to the period before the doctors’ strike, significantly fewer neonates were admitted during all time periods, with the lowest average number during the nurses’ strike (28, 95% CI 23–34, *p* < 0.01). There was no evidence of a difference in numbers of admissions of HIV-exposed versus HIV-unexposed neonates (all interaction *p*-values > 0.22), Fig. 1. Admissions by time period are presented in Supplementary Table 1.

### Indicator 2: neonatal mortality

**Fig. 2:**
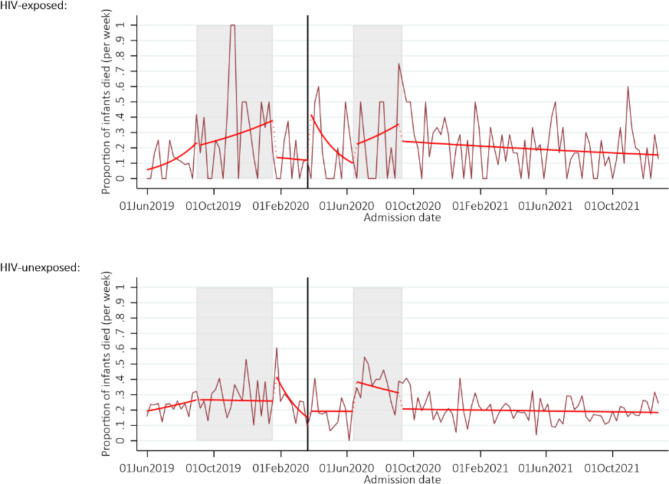
Neonatal mortality by HIV exposure. *Note:* The grey shaded areas represent the doctors and nurses strike, and the solid black vertical line represents the start of the COVID-19 pandemic in Zimbabwe. The maroon line shows the proportion of neonates dying each week, and the red line the number from the fitted model allowing a change in slope and level in each time period

Across the whole study period, among those with matched outcome data, 1,509 (21%) neonates died. There was no evidence of change in mortality at the start of the pandemic (RR 1.06, 95% CI 0.53–2.13, *p* = 0.87). During the nurses’ strike mortality increased to 34% (95% CI 25–46), however there was no change in the total number of deaths in this time period compared to pre-COVID times (Supplementary Fig. 1 and Supplementary Table 2). There was no evidence of an increase in mortality in HIV-exposed neonates compared to HIV-unexposed neonates (all interaction *p*- values > 0.39), Fig. 2. Case fatality rates by time period are presented in Supplementary Table 3.

### Indicator 3: HIV testing

**Fig. 3:**
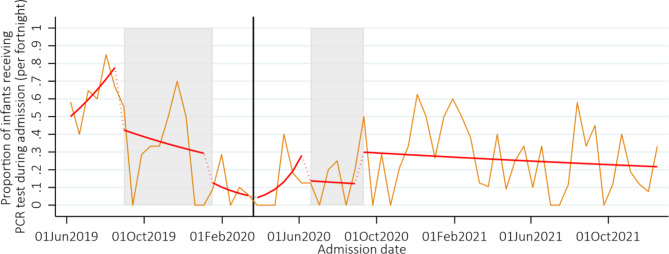
Proportion of HIV-exposed neonates who received a HIV PCR test during admission. *Note:* The grey shaded areas represent the doctors and nurses strike, and the solid black vertical line represents the start of the COVID-19 pandemic in Zimbabwe. The orange line shows the proportion of neonates who received a HIV PCR test each week, and the red line the number from the fitted model allowing a change in slope and level in each time period

Pre-pandemic, 64% (95% CI 47–87) of HIV-exposed neonates received a PCR test, this proportion dropped significantly during all subsequent time periods ranging from 8 to 36%. Fewer HIV-exposed neonates received a PCR test during the pandemic (23%) compared to the pre-pandemic periods (40%) (RR 0.59, 95% CI 0.41–0.84, *p* < 0.01), Fig. 3, Supplementary Table 4. Across the whole time-period only ten neonates received a positive PCR test by discharge; numbers were too small to consider trends over time.

### Indicator 4: HIV prophylaxis

**Fig. 4:**
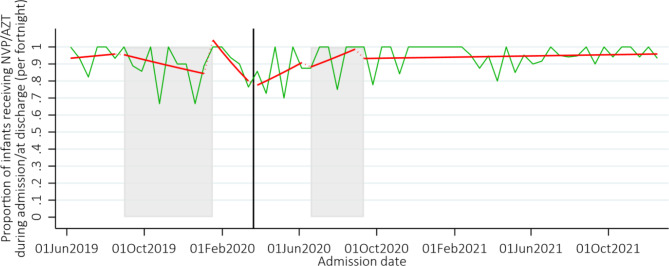
Proportion of HIV-exposed neonates who received ARV prophylaxis during admission. *Note:* The grey shaded areas represent the doctors and nurses strike, and the solid black vertical line represents the start of the COVID-19 pandemic in Zimbabwe. The green line shows the proportion of neonates who received ARV prophylaxis each week, and the red line the number from the fitted model allowing a change in slope and level in each time period

The proportion of HIV-exposed neonates who received antiretroviral prophylaxis during admission was high throughout, averaging between 84% and 95% in each time-period, Fig. 4, Supplementary Table 5. Over the whole time-period, of the 904 HIV-exposed neonates, 379 (42%) were classified as low-risk, 108 (12%) as high risk and 417 (46%) were not classified.

There was an increase in proportion of unclassified neonates during the pandemic (*p* < 0.01); amongst neonates with known status there was no change in risk (*p* = 0.74), Table 2.

**Table 2:** Classification of HIV-exposed neonates as high/low risk, before and during the pandemic

HIV exposed neonates risk categorisation	*Before*	*During*	*Overall*
	n (% of total; % excluding unknown)		
**Low**	133 (52%; 79%)	246 (38%; 77%)	379 (42%; 78%)
**High**	36 (14%; 21%)	72 (11%; 23%)	108 (12%; 22%)
**Unknown**	89 (35%)	328 (51%)	417 (46%)

Over the whole time-period, of the neonates categorised as low risk, 77% (293) were commenced on the recommended regimen (NVP alone), of the neonates categorised as high risk, 70% (76) were commenced on the correct regimen (NVP and AZT). There was a significant drop in the proportion receiving the correct regimen among the low-risk neonates (*p* = 0.02) during the pandemic, and some evidence of a drop amongst the high-risk neonates (*p* = 0.10), Table 3.

**Table 3:** Proportion of HIV-exposed neonates receiving the recommended antiretroviral regimen per risk category

HIV exposed neonates risk categorisation	*Before*	*During*	*Overall*
	n (%)		
**Low**	112 (84%)	181 (74%)	293 (77%)
**High**	29 (81%)	47 (65%)	76 (70%)

## Discussion

This study is the only detailed analysis of the impact of COVID-19 on neonatal outcomes for HIV-exposed neonates to our knowledge. Despite predicted concerns of increases in both numbers of HIV-exposed neonates and increased mortality in those exposed, our data for hospital-admitted neonates, has not shown this to be the case. Our findings suggest there is some resilience in the PMTCT programme in Zimbabwe, despite the challenges the COVID-19 pandemic has placed upon the health system.

The time-period reviewed was characterised by three COVID-19 “waves” with three associated national level lockdowns, the beginning of the fourth wave and combined with two separate periods of HCP industrial action. The relative reduction in admissions was similar among HIV-exposed and unexposed neonates, suggesting both groups were similarly impacted by COVID-19. However, it is possible that any reduction in the number of HIV-exposed neonates caused by reduced maternal testing might not be seen immediately, due to the 9-month gestation period. The fact that the absolute numbers of deaths did not increase despite decreases in the total number of admissions suggests that the sickest babies were still attending and admitted to the neonatal unit as needed. The reduction in total admissions is consist with trends before and during the initial few months of the pandemic at the same tertiary facility, alongside a Malawian tertiary neonatal unit [13]. The available evidence demonstrates the detrimental impact on health service delivery during the COVID-19 pandemic in low-resource settings [17]. Specifically in Zimbabwe, Bikwa et al. reported a reduction in maternal and neonatal service delivery, during the first COVID-19 wave, resulting in an increased risk of stillbirths and neonatal deaths [18]. Many recent publications have described the disruption to HIV services but not specifically the PMTCT programme and the impact on HIV-exposed neonates [19].

We reviewed the impact on HIV-exposed high-risk neonates to explore whether with PMTCT disruption, there would be an increase in the number of high-risk HIV-exposed neonates. Although we found no evidence of this, there was an increase in the number who were not risk-stratified. This could be due to several reasons, for example reduced senior clinical oversight due to the increased work demands and reduced staffing associated with the pandemic waves [20], the junior HCPs may have lacked the confidence to accurately stratify each neonate. Alongside this the time pressure to admit and discharge neonates may have meant the information to accurately stratify the HIV-exposed neonates may not have been available or accurately recorded. This in turn could have impacted the initiation of the correct treatment regime. The importance of this accurate diagnosis is essential as HIV disease can advance rapidly in infants, with a peak mortality rate at two to three months of age, therefore it is vital for EID to commence appropriate ART to halt this progression.

The number of HIV-exposed neonates receiving antiretroviral prophylaxis during admission remained high throughout the time-period reviewed. Neonates who did not receive prophylaxis during admission may have received it elsewhere after discharge, from community HIV clinics and pharmacy programmes; this data would not be captured by the Neotree dataset.

The COVID-19 pandemic did appear to have an impact on the number of neonates who had PCR-testing during admission. The data have also shown, within Sally Mugabe Central Hospital, the number of neonates requiring initiation of antiretroviral prophylaxis was unaffected remaining at pre-COVID levels (84–95%). This is in keeping with data from an interrupted time series analysis of 65 primary care clinics in South Africa during the 2020 COVID-19 lockdown which reported a significant impact on HIV testing but ART provision was maintained [21].

The strengths of this study include the use of one of the few in-depth data sets collected from a neonatal unit in a low-resource setting throughout the duration of the COVID-19 pandemic. However, there are several limitations to the dataset. Firstly, the numbers of admissions were too small to accurately assess the proportions of low and high-risk HIV-exposed neonates by each time-period. Within the Neotree application the HCP is required to decide if the neonate is high or low risk; this is not a mandatory field and so if they were unsure, with insufficient clinical oversight, they are able to class as “HIV-exposed” with no classification of risk. Secondly, within Neotree clinicians are asked to report if PCR testing was conducted or antiretroviral prophylaxis was given, and where not reported it was assumed these did not occur, which may have led to underestimation across all time periods. Thirdly, this dataset may have missed mothers who delivered at home, who may have had poorer outcomes and therefore we may have missed the neonates at highest risk. This dataset only captures facility-based deliveries.

This data complements the findings of the wider mixed-methods study (ClinicalTrials.gov: NCT04782739) including the programme and population level analysis, alongside the qualitative data capturing the detailed experiences of pregnant mothers seeking healthcare during this time. The programme and population level analysis highlighted the biggest impact of the COVID-19 pandemic was on maternal HIV testing, with most programme level indicators rapidly recovering over time, but a slower recovery seen at a population level [14]. The qualitative interviews reported that the clinic closures significantly impacted the delivery of PMTCT services and highlighted that despite the persistence of the mothers to access antenatal and PMTCT service care, essential services were not delivered.

## Conclusion

Despite the challenges the COVID-19 pandemic has placed upon health systems globally, our findings would suggest there is some resilience in the PMTCT programme in Zimbabwe. While antiretroviral prophylaxis for identified HIV-exposed neonates remained high throughout, concerning data on low admissions and increased mortality risks, similar in HIV-exposed and unexposed neonates, and reduced HIV testing, suggest some aspects of care may have been compromised due to the indirect effects of the COVID-19 pandemic. Identifying these weaknesses within neonatal health care services can be used to develop strategies to be resilient to these challenges and to ensure Zimbabwe stays on track to eliminate mother-to-child-transmission by 2025, as per the Zimbabwe National HIV and AIDS Strategic Plan 2021–2025 [22]. Further review of the impact of other external crises would be useful in the development of health system and programme strategies to prepare for these challenges and the likelihood of future pandemics. Quantifying and understanding the indirect impact on mothers and infants with co-morbidities and co-infections such as HIV, malaria and TB is vital to maintain the gains that have been made over the last two decades, to inform clinical practice and public policy makers.

### Electronic supplementary material

Below is the link to the electronic supplementary material.

Supplementary Material 1

Supplementary Material 2

Supplementary Material 3

Supplementary Material 4

Supplementary Material 5

Supplementary Material 6

## Data Availability

The datasets generated and analysed during the current study are not publicly available as the database is under development and subject to ongoing negotiation with relevant Ministries of Health but are available from the corresponding author on reasonable request.

## List of abbreviations

ART: Antiretroviral therapy
AZT: Zidovudine
CI: Confidence interval
COVID-19: Coronavirus diseases 2019
EID: Early infant diagnosis
HCP: Healthcare provider
HIV: Human immunodeficiency virus
LRS: Low resource settings
NNU: Neonatal unit
NVP: Nevirapine
PCR: Polymerase chain reaction
PMTCT: Prevention of mother to child transmission
