# Supplementary figures and images for: Effects of the COVID-19 pandemic on the outcomes of HIV-exposed neonates: a Zimbabwean tertiary hospital experience
Source: BMC Pediatr. 2024 Jan 5;24:16. doi: 10.1186/s12887-023-04473-5 (PMC10768266; doi:10.1186/s12887-023-04473-5)

***Supplementary Figure 1: Number of deaths over time***


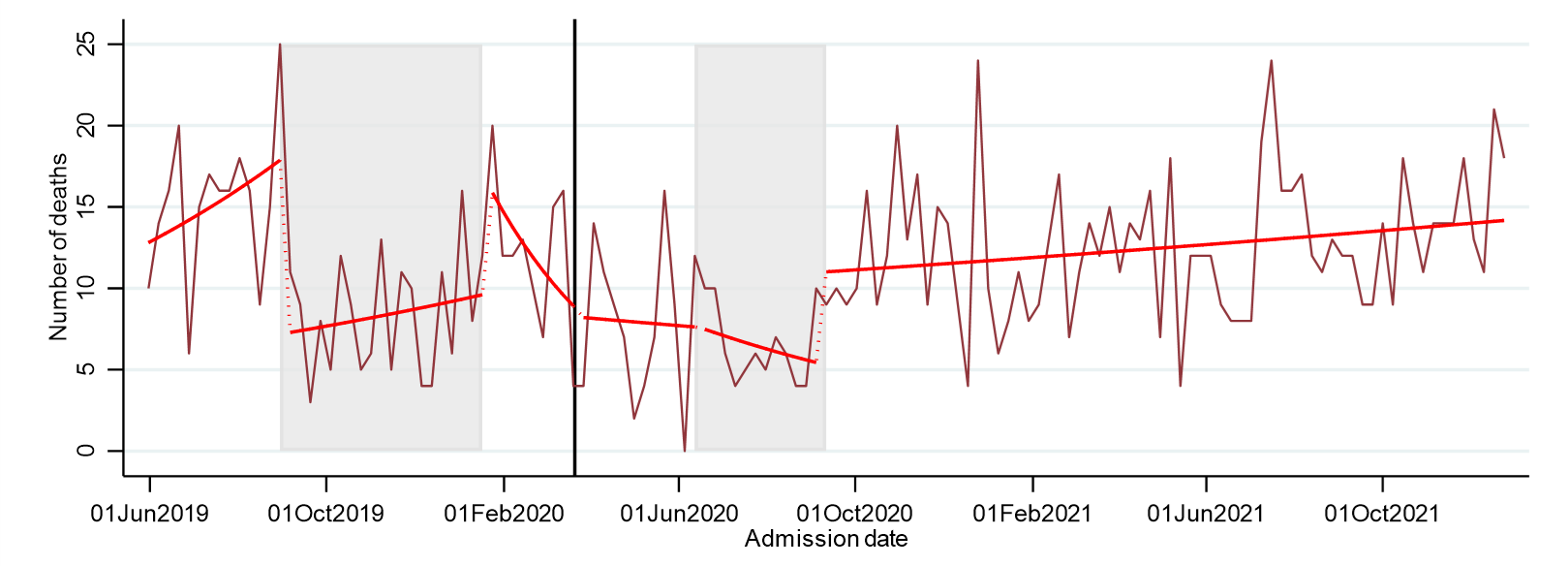

Supplement: Supplementary file 1 — Supplementary Material 1 [file 12887_2023_4473_MOESM1_ESM.docx]
