# Supplementary material for: Effects of the COVID-19 pandemic on the outcomes of HIV-exposed neonates: a Zimbabwean tertiary hospital experience
Source: BMC Pediatr. 2024 Jan 5;24:16. doi: 10.1186/s12887-023-04473-5 (PMC10768266; doi:10.1186/s12887-023-04473-5)
Supplement: Supplementary file 2 — Supplementary Material 2 [file 12887_2023_4473_MOESM2_ESM.docx]

***Supplementary Table 1a, 1b and 1c: Comparison of admissions by HIV exposure across each time period***

***Table 1a: Number of admissions overall***

|  | Before doctor’s strike | Doctor’s strike | Doctors strike to COVID | COVID to nurses strike | Nurses strike | After nurses strike |
| --- | --- | --- | --- | --- | --- | --- |
| Number admitted (95% CI), p-value (vs. before doctors strike) | 78.2 (70.2, 87.1) | 35.3 (30.8, 40.3), p<0.001 | 61.2 (53.1, 71.8), p=0.013 | 54.8 (47.7, 62.9), p<0.001 | 27.7 (22.8, 33.6), p<0.001 | 73.4 (69.7, 77.1), p=0.29 |
| Change per week, RR (95% CI) | 0.99 (0.97, 1.02), p=0.65 | 1.01 (0.99, 1.04), p=0.29 | 1.07 (1.01, 1.13), p=0.029 | 1.02 (0.98, 1.06), p=0.37 | 0.98 (0.93, 1.04), p=0.57 | 1.00 (1.00, 1.01), p=0.002 |
| Change at start of period vs. end of last, RR (95% CI) | - | 0.42 (0.30, 0.58), p<0.001 | 1.20 (0.82, 1.75), p=0.35 | 0.72 (0.49, 1.07), p=0.11 | 0.50 (0.33, 0.76), p=0.001 | 2.53 (1.74, 3.69), p<0.001 |

***Table 1b: Number of admissions: HIV-exposed neonates***

|  | Before doctor’s strike | Doctor’s strike | Doctors strike to COVID | COVID to nurses strike | Nurses strike | After nurses strike |
| --- | --- | --- | --- | --- | --- | --- |
| Number admitted (95% CI), p-value (vs. before doctors strike) | 8.5 (7.0, 10.4) | 4.4 (3.4, 5.5), p<0.001 | 6.7 (5.0, 8.8), p=0.17 | 5.8 (4.4, 7.5), p=0.021 | 3.3 (2.4, 4.7), p<0.001 | 7.8 (7.1, 8.6), p=0.435 |
| Change per week, RR (95% CI) | 1.02 (0.96, 1.09), p=0.44 | 1.00 (0.95, 1.05), p=0.99 | 1.10 (0.95, 1.27), p=0.20 | 1.03 (0.94, 1.14), p=0.62 | 1.04 0.91, 1.18), p=0.56 | 1.01 (1.00, 1.01), p=0.10 |
| Change at start of period vs. end of last, RR (95% CI) | - | 0.44 (0.21, 0.92), p=0.029 | 1.02 (0.40, 2.63), p=0.96 | 0.62 (0.23, 1.68), p=0.34 | 0.40 (0.14, 1.15), p=0.089 | 1.59 (0.70, 3.64), p=0.27 |

***Table 1c: Number of admissions: HIV unexposed neonates***

|  | Before doctor’s strike | Doctor’s strike | Doctors strike to COVID | COVID to nurses strike | Nurses strike | After nurses strike |
| --- | --- | --- | --- | --- | --- | --- |
| Number admitted (95% CI), p-value (vs. before doctors strike) | 69.7 (62.5, 77.7) | 30.9 (27.0, 35.4), p<0.001 | 55.1 (47.3, 64.2), p=0.014 | 49.0 (42.6, 56.4), p<0.001 | 24.3 (19.9, 29.7), p<0.001 | 65.6 (62.3, 69.0), p=0.32 |
| Change per week, RR (95% CI) | 0.99 (0.97, 1.01), p=0.39 | 1.01 (0.99, 1.03), p=0.16 | 1.06 (1.01, 1.11), p=0.017 | 1.02 (0.98, 1.05), p=0.34 | 0.98 (0.93, 1.02), p=0.34 | 1.00 (1.00, 1.01), p=0.001 |
| Change at start of period vs. end of last, RR (95% CI) | - | 0.41 (0.31, 0.55), p<0.001 | 1.22 (0.88, 1.69), p=0.23 | 0.74 (0.53, 1.03), p=0.077 | 0.51 (0.36, 0.74), p<0.001 | 2.70 (1.94, 3.76), p<0.001 |
