# Supplementary material for: Effects of the COVID-19 pandemic on the outcomes of HIV-exposed neonates: a Zimbabwean tertiary hospital experience
Source: BMC Pediatr. 2024 Jan 5;24:16. doi: 10.1186/s12887-023-04473-5 (PMC10768266; doi:10.1186/s12887-023-04473-5)
Supplement: Supplementary file 3 — Supplementary Material 3 [file 12887_2023_4473_MOESM3_ESM.docx]

***Supplementary Table 2: Comparison of number of deaths across each time period***

|  | Before doctor’s strike | Doctor’s strike | Doctors strike to COVID | COVID to nurses’ strike | Nurses strike | After nurses’ strike |
| --- | --- | --- | --- | --- | --- | --- |
| Number dying (95% CI), p-value (vs. before doctors strike) | 15.2 (12.9, 18.0) | 8.4 (6.9, 10.3), p<0.01 | 12.1 (9.4, 15.6), p=0.12 | 7.9 (6.1, 10.3), p<0.01 | 6.4 (4.8, 8.6), p<0.01 | 12.5 (11.5, 13.7), p=0.04 |
