# Supplementary material for: Effects of the COVID-19 pandemic on the outcomes of HIV-exposed neonates: a Zimbabwean tertiary hospital experience
Source: BMC Pediatr. 2024 Jan 5;24:16. doi: 10.1186/s12887-023-04473-5 (PMC10768266; doi:10.1186/s12887-023-04473-5)
Supplement: Supplementary file 4 — Supplementary Material 4 [file 12887_2023_4473_MOESM4_ESM.docx]

***Supplementary Table 3a, 3b and Table 3c: Comparison of case fatality rates by HIV exposure across each time period***

***Table 3a: Case fatality rate overall***

|  | Before doctor’s strike | Doctor’s strike | Doctors strike to COVID | COVID to nurses strike | Nurses strike | After nurses strike |
| --- | --- | --- | --- | --- | --- | --- |
| Proportion dying (95% CI), p-value (vs. before doctors strike) | 22% (19%, 25%) | 27% (22%, 32%), p=0.091 | 24% (19%, 30%), p=0.45 | 19% (15%, 25%), p=0.46 | 34% (26%, 44%), p=0.004 | 19% (18%, 21%), p=0.23 |
| Change per week, RR (95% CI) | 1.03 (0.99, 1.07), p=0.16 | 1.00 (0.98, 1.03), p=0.88 | 0.89 (0.81, 0.97), p=0.006 | 0.98 (0.92, 1.06), p=0.65 | 0.99 (0.92, 1.05), p=0.69 | 1.00 (0.99, 1.00), p=0.27 |
| Change at start of period vs. end of last, RR (95% CI) | - | 1.01 (0.66, 1.55), p=0.97 | 1.42 (0.88, 2.29), p=0.15 | 1.06 (0.53, 2.13), p=0.87 | 2.06 (1.09, 3.87), p=0.025 | 0.67 (0.41, 1.09), p=0.11 |

***Table 3b: Case fatality of HIV exposed neonates***

|  | Before doctor’s strike | Doctor’s strike | Doctors strike to COVID | COVID to nurses strike | Nurses strike | After nurses strike |
| --- | --- | --- | --- | --- | --- | --- |
| Proportion dying (95% CI), p-value (vs. before doctors strike) | 13% (9%, 21%) | 29% (20%, 42%), p=0.008 | 13% (7%, 24%), p=0.90 | 22% (13%, 36%), p=0.17 | 29% (16%, 52%), p=0.037 | 19% (16%, 23%), p=0.15 |
| Change per week, RR (95% CI) | 1.05 (0.87, 1.27), p=0.59 | 1.03 (0.97, 1.09), p=0.40 | 0.95 (0.67, 1.34), p=0.75 | 0.89 (0.73, 1.09), p=0.26 | 1.09 (0.91, 1.30), p=0.34 | 0.98 (0.97, 1.00), p=0.018 |
| Change at start of period vs. end of last, RR (95% CI) | - | 3.21 (0.79, 13.02), p=0.10 | 0.25 (0.04, 1.53), p=0.13 | 5.34 (0.83, 34.36), p=0.078 | 2.18 (0.31, 15.53), p=0.44 | 0.43 (0.15, 1.22), p=0.11 |

***Table 3c: Case fatality of HIV unexposed neonates***

|  | Before doctor’s strike | Doctor’s strike | Doctors strike to COVID | COVID to nurses strike | Nurses strike | After nurses strike |
| --- | --- | --- | --- | --- | --- | --- |
| Proportion dying (95% CI), p-value (vs. before doctors strike) | 23% (19%, 27%) | 26% (22%, 32%), p=0.27 | 26% (20%, 32%), p=0.41 | 19% (15%, 25%), p=0.28 | 35% (26%, 46%), p=0.01 | 19% (18%, 21%), p=0.10 |
| Change per week, RR (95% CI) | 1.03 (0.91, 1.18), p=0.62 | 0.99 (0.93, 1.05), p=0.74 | 0.82 (0.64, 1.05), p=0.12 | 0.94 (0.77, 1.15), p=0.55 | 0.99 (0.89, 1.11), p=0.91 | 0.99 (0.98, 1.00), p=0.13 |
| Change at start of period vs. end of last, RR (95% CI) | - | 2.44 (0.74, 8.06), p=0.14 | 1.36 (0.41, 4.47), p=0.62 | 1.76 (0.27, 11.66), p=0.56 | 6.81 (1.44, 32.35), p=0.016 | 0.22 (0.09, 0.57), p=0.002 |
