# Supplementary material for: Effects of the COVID-19 pandemic on the outcomes of HIV-exposed neonates: a Zimbabwean tertiary hospital experience
Source: BMC Pediatr. 2024 Jan 5;24:16. doi: 10.1186/s12887-023-04473-5 (PMC10768266; doi:10.1186/s12887-023-04473-5)
Supplement: Supplementary file 5 — Supplementary Material 5 [file 12887_2023_4473_MOESM5_ESM.docx]

***Supplementary Table 4: Comparison of HIV-exposed neonates who received a HIV PCR test during admission across each time-period***

|  | Before doctor’s strike | Doctor’s strike | Doctors strike to COVID | COVID to nurses strike | Nurses strike | After nurses strike |
| --- | --- | --- | --- | --- | --- | --- |
| Proportion tested (95% CI), p-value (vs. before doctors strike) | 64% (47, 87) | 36% (24, 56), p=0.04 | 8% (3, 24), p<0.01 | 14% (6, 34), p<0.01 | 13% (4, 42), p=0.01 | 25% (20, 31), p<0.01 |
| Change per fortnight, RR (95% CI) | 1.09 (0.90, 1.33), p=0.38 | 0.96 (0.83, 1.11), p=0.58 | 0.81 (0.38, 1.72), p=0.58 | 1.46 (0.77, 2.77), p=0.25 | 0.98 (0.50, 1.90), p=0.94 | 0.99 (0.97, 1.01), p=0.43 |
| Change at start of period vs. end of last, RR (95% CI) | - | 0.55 (0.23, 1.27), p=0.16 | 0.43 (0.06, 3.08), p=0.40 | 0.51 (0.03, 7.60), p=0.63 | 0.23 (0.01, 5.33), p=0.36 | 2.46 (0.30, 20.32), p=0.40 |
