# Supplementary material for: Effects of the COVID-19 pandemic on the outcomes of HIV-exposed neonates: a Zimbabwean tertiary hospital experience
Source: BMC Pediatr. 2024 Jan 5;24:16. doi: 10.1186/s12887-023-04473-5 (PMC10768266; doi:10.1186/s12887-023-04473-5)
Supplement: Supplementary file 6 — Supplementary Material 6 [file 12887_2023_4473_MOESM6_ESM.docx]

***Supplementary Table 5: HIV-exposed neonates who received ARV prophylaxis during admission across each time period***

|  | Before doctor’s strike | Doctor’s strike | Doctors strike to COVID | COVID to nurses strike | Nurses strike | After nurses strike |
| --- | --- | --- | --- | --- | --- | --- |
| Proportion receiving prophylaxis (95% CI), p-value (vs. before doctors strike) | 93% (90,96) | 91% (85, 96), p=0.54 | 90% (84, 97), p=0.51 | 84% (78, 91), p=0.04 | 94% (85, 103), p=0.85 | 95% (92, 97), p=0.31 |
| Change per fortnight, RR (95% CI) | 1.00 (1.00, 1.01), p=0.42 | 0.99 (0.97, 1.01), p=0.16 | 0.94 (0.89, 0.98), p=0.01 | 1.03 (0.99, 1.08), p=0.18 | 1.02 (0.97, 1.08), p=0.40 | 1.00 (1.00, 1.00), p=0.53 |
| Change at start of period vs. end of last, RR (95% CI) | - | 1.01 (0.90, 1.13), p=0.90 | 1.23 (1.04, 1.46), p=0.01 | 0.85 (0.72, 1.00), p=0.05 | 0.91 (0.70, 1.20), p=0.51 | 0.94 (0.80, 1.11), p=0.50 |
